# Forecasting head lice (*Pediculidae*: *Pediculus humanus capitis*) infestation incidence hotspots based on spatial correlation analysis in Northwest Iran

**DOI:** 10.14202/vetworld.2020.40-46

**Published:** 2020-01-06

**Authors:** Davoud Adham, Eslam Moradi-Asl, Malek Abazari, Abedin Saghafipour, Parisa Alizadeh

**Affiliations:** 1 Department of Public Health, School of Public Health, Ardabil University of Medical Sciences, Ardabil, Iran; 2 Arthropod Borne Diseases Research Center, Ardabil University of Medical Sciences, Ardabil, Iran; 3 Department of Public Health, Faculty of Health, Qom University of Medical Sciences, Qom, Iran

**Keywords:** head lice infestation, Iran, *Pediculus humanus capitis*, spatial analysis

## Abstract

**Background and Aim:**

*Pediculus humanus capitis* has been prevalent throughout the world, especially in developing countries among elementary students and societies with a weak socio-economic status. This study aimed to forecast head lice (*Pediculidae*: *P. capitis*) infestation incidence hotspots based on spatial correlation analysis in Ardabil Province, Northwest Iran.

**Materials and Methods:**

In this retrospective analytical study, all cases of head lice infestations who were confirmed by Centers for Disease Control office have been studied from 2016 to 2018. Head lice infestation incidence hotspots in the province should be detected based on general G statistics in ArcMap GIS10.4.1. Furthermore, MaxEnt.3.3.3 model was used for modeling the high-risk areas.

**Results:**

The prevalence rate of pediculosis was 14.90/100,000 populations. The general G statistics revealed that the head lice infestation in this study area has a high cluster pattern. The analysis showed that the Parsabad and Germi counties were identified as a head lice infestation incidence hotspots. Statistical and spatial analyses of head lice infestation incidence showed a significant positive correlation with head lice infestation incidence hotspots and the altitudes (15-500 m), annual temperature range (14-16.5°C), and slope and average diurnal temperature (12-18°C).

**Conclusion:**

The results of this study showed that the most ecologically suitable areas of head lice occurrence were identified in two hotspots (Parsabad and Germi) in the Northern areas of Ardabil Province (Parsabad and Germi counties); in the borderline of Iran and the Republic of Azerbaijan.

## Introduction

The relationship between humans and head lice as one of the oldest insects probably started around more than 100,000 years ago. The first evidence of the presence of head lice in humans dwelling is related to head lice nit on head hair and was dated to 8000 B.C. [1]. Human head lice infestation (pediculosis) usually defined as an infestation of the hairy parts of the body with the human head-and-body louse. In this health issue, head lice are blood-feeding on the human several times a day and the lice saliva with medically important toxins is repeatedly injected into the body, therefore, its toxic effects appear in infested individuals including: Fatigue, irritability, pessimism, and feeling lazy mode [2]. Pediculosis has World Wise Distribution, but in temperate regions, its level of harassment is comparable to mosquitoes [3]. Head lice is prevalent in developing countries, and especially in primary schools students age groups and societies with a poor socioeconomic status [4-6].

The climate, geographical environment, health conditions, income, and family size are important factors affecting the prevalence of head lice [7-10]. Previously, many studies have been conducted in the world on the prevalence and factors affecting on head lice. For example, the prevalence rate in the Netherlands, Brazil, Turkey, and Venezuela was 4.8%, 35%, 1.2%, and 28.8%, respectively [11]. In different regions of Iran, the prevalence rate has been differently reported, including 4% in Urmia [11], 13.5% in Hamedan [12], 1.8% in Kerman [13], 4.7% in Sanandaj [14], and 10.20% in Ardabil Provinces [10]. The geographic information system (GIS) is a new tool used in vector-borne diseases (VBDs) studies, such as pediculosis and has led to significant changes in data interpretation and decision. Nowadays, researchers are now able to know the global distribution of the disease and its extent faster and identify the high-risk area by GIS software. This software can helps to a quick assessment of endemic areas, the accurate and reliable estimates of the population at risk and prediction of the distribution of the disease in areas that lack information or are impassable to determine the appropriate strategy for controlling and preventing of disease in these areas [15,16].

Considering the increasing trend of head lice infestation in Ardabil Province, this study was designed to forecast head lice (*Pediculidae*: *Pediculus humanus capitis*) infestation incidence hotspots based on spatial correlation analysis in Ardabil Province, Northwest Iran.

## Materials and Methods

### Ethical approval

Ethical clearance was earned from the Institutional Ethics Committee of Ardabil University of Medical Sciences. The ethics code for this project is IR.ARUMS.REC. 1396.16.

### Study area

**Figure-1:**
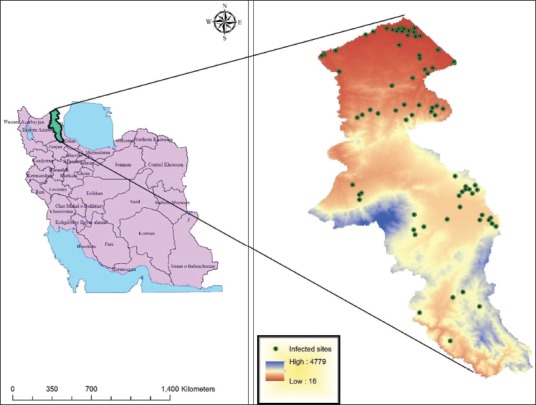
Geographical location of Ardabil Province and the sites of highest infestation of *Pediculus humanus capitis* Ardabil Province, Northwest of Iran (2016-2018).

Ardebil Province is located in the Northwest of Iran with geographical coordinates of 38.4853°N and 47.8911°E, which in the borderline the Republic of Azerbaijan. The province is mountainous in central and south parts and textured plains in the north that the highest point is Sabalan mount with an altitude of 4811 m and the lowest point of the Moghan plain with an altitude of 15 m above sea level. According to the latest census in 2016, its population is 1,270,420 people and its area is 17953 km^2^, which makes up 1% of the total area of Iran (Figure-1).

### Study design and data collection

This is a retrospective-analytical study and all information on humans head lice infestation was confirmed by the Centers for Disease Control office and collected from the health centers in 2016-2018. All data were classified based on age, gender, place, and time of the head lice infested cases. The prevalence rate of pediculosis in different years was estimated by the Statistical Package for the Social Sciences (SPSS) software version 23 (IBM, Chicago, USA).

### Ecological niche modeling

To determine the ecological niche of head lice, geographical coordinates of 75 areas (15 urban and 60 rural areas) were extracted that the over 50 human cases were infested with head lice and every year, cases of the head lice infestation are reported from these regions. MaxEnt.3.3.3 software, (Maximum Entropy) was applied for modeling the high-risk areas under head lice infestation. The MaxEnt software is based on the maximum entropy approach for modeling species niches and distributions. From a set of environmental grids and georeferenced occurrence localities, the model expresses a probability distribution where each grid cell has predicted suitability of conditions for the species. Nineteen climate and environmental variables were downloaded from the WorldClim database with a resolution of 1 km^2^. (http://www.worldclim.org/bioclim, version 1.4). The vegetation information normalized difference vegetation index of the region was also used for moderate resolution imaging spectroradiometer satellite imagery. The relationship between climate and environment variables with head lice was analyzed by the jackknife test. The jackknife is a resampling technique especially useful for variance and bias estimation. The jackknife estimator of a parameter is found by systematically leaving out each observation from a dataset and calculating the estimate and then finding the average of these calculations. Output results in different layers using ArcMap 10.4.1 software (Esri’s ArcGIS).

### Spatial autocorrelation

To perform spatial autocorrelation analysis of the data, they obtained the spatial data bank in the ArcGIS10.4.1 software and distribution maps of the disease cases in different years were drawn in the ArcMap environment. Spatial autocorrelation and determine of high/low clustering of pediculosis cases in the different counties of the study area were estimated. The spatial autocorrelation tool in ArcGIS measures spatial autocorrelation based on both feature locations and feature values simultaneously. Given our pediculosis cases data and the associated attribute (country border), we evaluated the pattern of the disease (clustered, dispersed, or random). General G value was calculated, and as well, both a score and p<0.05 were also calculated and were used to evaluate the significance of the index. Thus, the index is given as:


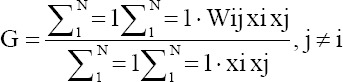


## Results

### Prevalence of *P. capitis*

The total number of people infested with head lice was 57,372 people in Ardabil Province. Out of the infested people, 14,428, 20,522, and 22,422 people were reported in 2016, 2017, and 2018 respectively. The prevalence rate of head lice in Ardabil Province’s population was averaging 14.90/100,000 which increased from 11.32/100,000 in 2016 to 17.18/100,000 in 2018. The prevalence rates in the two northern counties of the province including Parsabad (42.28%) and Germi (28%) were the most prevalent in 2018 (Table-1). A total, 88% of infested humans were females and the rest were males. Most of the cases (20%) were in the 6-17 year old age group who were students and the lowest cases were reported in the over 30 years age group (1.32%). The highest infestation rate was occurred in autumn (64.50%) and the lowest rate was recorded in spring (4.50%).

**Table-1:** Prevalence of pediculosis in Ardabil Province, Northwest of Iran (2016-2018).

Year	2016		2017		2018	
			
County	No.	Prevalence rate (×1000)	No.	Prevalence rate (×1000)	No.	Prevalence rate (×1000)
Meshkinshahr	325	2.14	1851	12.34	2215	13.92
Namin	1252	20.54	2514	41.45	526	8.76
Khalkhal	317	3.59	588	6.77	625	7.28
Sareyn	304	16.93	328	18	336	17.98
Nir	77	2.68	276	9.74	328	11.54
Kawsar	225	9.67	360	16.26	336	15.54
Germi	790	10	1066	13.86	2115	28
Ardabil	5996	10.03	5293	8.73	7250	11.8
Parsabad	4298	24.34	7233	40.72	7544	42.28
Bilehsavar	844	16.19	1013	19.7	1147	22.51
Total	14428	11.32	20522	16.05	22422	17.34

### Spatial analysis

**Figure-2:**
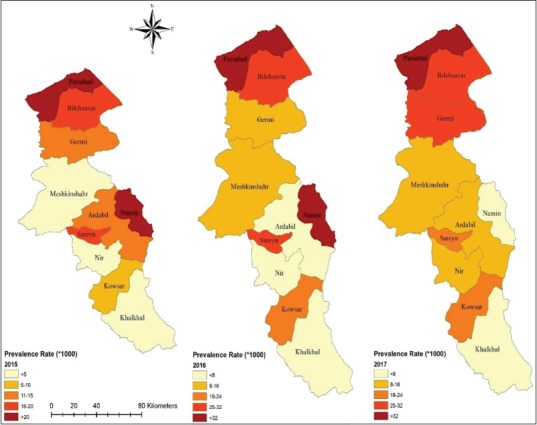
The prevalence of pediculosis in Ardabil Province, Northwest of Iran (2016-2018).

*P. capitis* infestation has been reported from all counties of Ardabil Province. The highest and lowest prevalence of pediculosis have been reported from Parsabad (35.87/1000) and Khalkhal (5.88/1000) counties during the past 3 years. Out of the 1695 villages with permanent residents, lice infestation was occurred in 570 villages (33.60%) and out of 26 urban areas, 24 cities (92.3%) were infected (Figure-2). The most infected villages were reported from Parsabad (81%) and the least infected villages in the Germi (8%) (Table-2).

**Table-2:** The number of cities and villages infestation with head lice in Ardebil Province, Northwest of Iran (2016-2018).

County	No. city	No. infected city	Infected city (%)	No. villages	No. infected villages	Infected villages (%)
Meshkinshahr	6	6	100	273	41	15
Namin	3	2	66.66	91	42	46.15
Khalkhal	3	2	66.66	142	26	18.30
Sareyn	1	1	100	27	15	55.50
Nir	2	2	100	99	26	26.26
Kowsar	1	1	100	98	19	19.38
Germi	2	1	50	315	25	8
Ardabil	2	2	100	151	46	29.80
Parsabad	4	4	100	247	200	81
Bilehsavar	2	2	100	252	130	51.58
Total	26	24	88.88	1695	570	33.62

**Figure-3:**
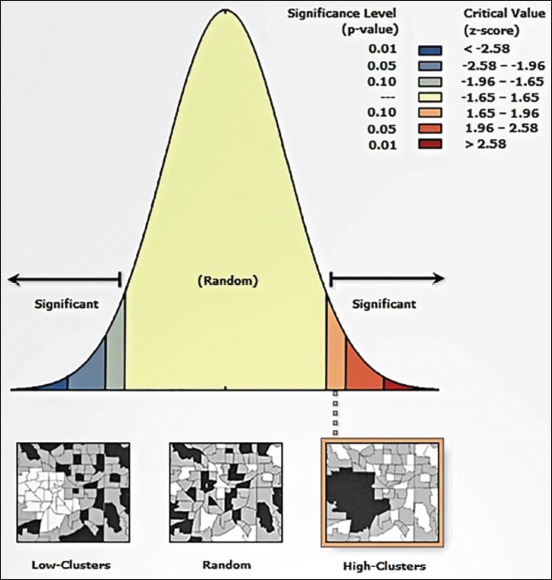
General G factor analysis of *Pediculus humanus capitis* cases in different counties of, Ardabil Province, Northwest of Iran (2016-2018).

The results of the general G analysis indicated that the hotspots of this health issue are located in the northern areas of the province (Parsabad and Germi). The high-low clustering report of the general G factor given the z-score of 5.04132446743, there is a <1% likelihood that this high-clustered pattern could be the result of random chance (p=0.0000). These areas with human head lice infested were bordered by the Republic of Azerbaijan; as a result, the risk of transmission of the *P. capitis* in these areas is higher than in other areas of the province (Figure-3).

### Modeling and suitable sites for head lice

**Figure-4:**
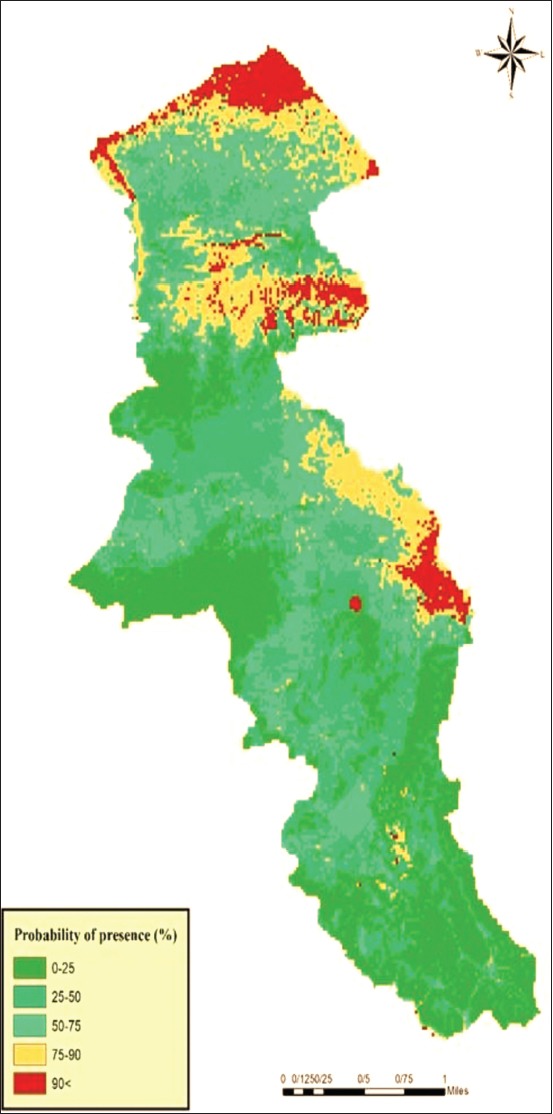
Ecologically suitable sites for head lice Ardabil Province, Northwest of Iran (2016-2018).

**Figure-5:**
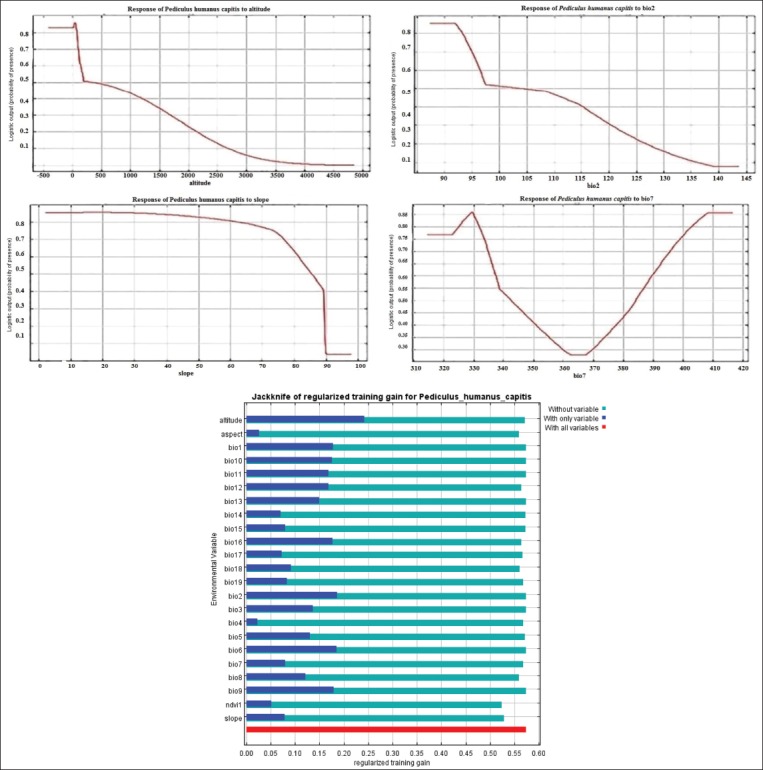
The results of jackknife test and the effect of important variables affecting pediculosis Ardabil Province, Northwest of Iran (2016-2018).

The results of MaxEnt model showed that the most ecologically suitable areas of pediculosis occurrence were identified in two hotspots (Figure-4) in Parsabad and Germi counties with a population of 313,837 at risk. The numbers under the ROC and AUC curves were 0.866 and 0.827, respectively, for training and testing data. The results of the jackknife test showed that the most important environmental and climate factors were affecting the prevalence of pediculosis in Ardabil province, including altitude, annual temperature range (Bio7), slope, and the mean diurnal range (Bio2) (Figure-5). The prevalence of pediculosis was occurrence at an altitude of between 15 and 1500 m above sea level.

## Discussion

In this study, the estimated average prevalence of pediculosis in Ardabil Province was 14.90/1000 during the past 3 years. In primary school students age group, the prevalence was 20%, and this showed that infestation in students was much higher than in other age groups. In other regions of Iran, the prevalence of pediculosis has been reported between 0.47% and 28.5%. Most studies have been conducted in primary schools [17-22]. Usually, the infestation rate of pediculosis in females is more than males, and in this study was more than 7 times, which can be due to the type of cover and hair size. This condition and factors have been reported in Iran and other countries [23-25]. According to the results of these previous studies, most infestations were reported in the fall season, which is in Iran at the same time as the starting of schools and the beginning of the cold season and changes in coverage between people. Moreover, in this season, the use of warm clothes, shawls and hats are increased and these are the factors to increase the infestation and transmission of head lice between students in schools [10,14,25].

The results of spatial autocorrelation of *P. capitis* infestation in Ardabil Province indicated that there was most distribution in counties located in the Northern areas of the province in the borderline of Iran and the Republic of Azerbaijan. The head lice infestation rate was very high in the urban and rural area in the Northern areas of Ardabil Province (Parsabad county) that over than 80% of rural areas were infested with head lice and the General G statistics revealed that the head lice infestation in this study area has a high cluster pattern.

The results showed that the pediculosis has been ascending in the past 3 years and in 2018 compared to 2016, more areas of the province have been infested, which is similar to other studies in different parts of the country [26-28]. The study on distribution and ecological niches of head lice have not been performed so far and this is the first study to be done using the MaxEnt model; however, many studies have been conducted on other species of parasites and vectors such as the vectors of visceral leishmaniasis (VL) in Iran and Ardabil Province. For example, the model for the vector of VL in Iran [29] as well as modeling for the causative agent of VL (*Leishmania infantum*) in Ardabil [26]. Modeling for determining high-risk areas under head lice infestation showed that the two regions in the Ardabil Province (Parsabad and Germi) were high-risk areas that these regions are located in the north of the province.

Based on the finding of the present study, out of 19 climates and environmental factors that used in modeling, the result of the model showed that four factors had the greatest impact on head lice distribution including altitude, annual temperature range, slope, and mean diurnal range. The altitude of the northern areas is 15-1500 m above sea level and whatever altitude is increasing above from 1500 m, the prevalence of pediculosis is reduced. In studies accrued on other vectors of diseases in different parts of the world and Iran, the altitude factor was the high impact on the distribution of species and the prevalence of the disease [27-28,30].

In Ardabil Province, where has been modeled as vectors of VL in 2018, the altitude factor had a large role in vector and disease distribution [26,30]. In this study, the most important factors that affected on the distribution of head lice were temperature that two factors the annual temperature range and mean diurnal range of the life cycle of the vector and prevalence of disease are affected by the development of a suitable environment for the growth and development of the vector [2]. In modeling studies for VBDs, temperature factors are critical factors for vectors growth and prevalence and incidence of diseases [31-34].

## Conclusion

According to the results of this study, the prevalence of pediculosis in Ardabil Province was higher among the students. The two hotspots and high-risk areas were identified in this province and in the distribution of pediculosis. In addition, climate and environmental factors (altitude and temperature) were very effective in the distribution of pediculosis in this area.

## Authors’ Contributions

EM and AS collected the data and drafted the manuscript. DA, EM, and AS analyzed the data and edited the manuscript. EM, PA, and MA designed the work with overall monitoring, analyzed the data and edited the manuscript. All authors drafted, read, and approved the final manuscript.
